# Address Delivered before the American Society of Dental Surgeons

**Published:** 1845-09

**Authors:** Eleazar Parmly

**Affiliations:** President.


THE AMERICAN

### JOURNAL OF DENTAL SCIENCE.

Vol. VI.] SEPTEMBER, 1845. [No. 1.

### ARTICLE I.

*Address delivered before the American Society of Dental Surgeons, at their Sixth Annual Meeting*, held in the City of New York. By ELEAZAR PARMLY, M. D., President.

*Gentlemen, and Respected Colleagues*
*of the American Society of Dental Surgeons:*

I am happy in having the pleasure of meeting with you, and of seeing so many of my professional brethren once more together, at the Annual Session of our highly valued Institution.

Having now completed thirty full years in professional study, and labor, it is to me a most gratifying sight, to see assembled around me, men from various parts of this country, who are well known for their general intelligence and personal worth, for their strict truth and moral integrity, and for their high attainments in professional learning and manual practice; particularly when I remember that at an early period of my professional life, I travelled from Philadelphia to New Orleans, through the western and southern states, without meeting but one dentist who had any pretensions to an acquaintance with the science or practice of Dental Surgery, and that one was A. J. Shymanski, a well educated Polish gentleman, of the highest respectability in his own country, and distinguished here by all who knew him, for great moral worth, refined intellect, amiable and correct deportment, and who was really the first and only dentist of gentlemanly and professional attainments, that I saw during that long journey, and such an one as was rarely met with in that day, away from the larger cities. And when I remember too, that it is but six years since the late venerable President of this Society, travelled from Baltimore to Boston, *without success,* in an attempt to arouse his professional brethren, to the importance of a national society, there being at that time a local one in this city, which had been for some time in successful operation, in a free interchange of views and opinions among its members, at their social meetings; and occasionally having lectures on the most important subjects, connected with dental practice. The local organization, as most of you are probably aware, became extinct, in the greater magnitude, superior advantages, and higher popularity of our favorite and fondly cherished Institution, in the same manner as the faint glimmering of the star of the morning becomes invisible in the brighter light of the rising sun.

This great and auspicious revolution has been effected by our united efforts, at the suggestion, and earnest, urgent instigation of one of our fathers, who is now asleep. The indefatigable Hayden, whose name I mention, not only with respect, but with feelings of veneration, lived to see his favorite Institution, in the full career of successful experiment, and when his days were numbered, he transmitted it to us and to posterity, for support.

He cherished the opinion that is entertained, I trust by all who hear me, that every important and useful profession among men, requires *organization,* by which is denoted a system of mutual instruction, protection, support and encouragement. The profession of the law, has its organization and instruction, in its *law schools,* its *legal journals, reports,* and associations. The interests of husbandmen, are cherished in their state and county agricultural societies, and by agricultural newspapers, and other periodicals, by which the present age is fortunately distinguished. The practitioners of general surgery and medicine, know well the advantages resulting from their medical societies, medical journals, and laws of professional etiquette.

All these, and other bodies of men, engaged in the same calling, have also the sanction and the protection of the laws. But it has fallen to the lot of dental surgery to be left wholly to its own *self-protection.* It would seem that legislatures, with a few honorable exceptions, prefer that their constituents should endure all the evils of quackery, rather than enact wholesome laws for their protection.

It is not a little remarkable, that the Spanish government, in its American colonies, and perhaps also in the mother country, pursue, in this respect, a much more enlightened policy, than our own boasted republic. There, a regular education and a diploma are just as necessary to dental as to medical practice, and those of our countrymen who have been there, have been obliged to go through a rigid examination.

In most of these United States, the community is left wholly uninformed, and unprotected in a department of surgical practice, which is in far greater demand, than what is called general surgery.

The objects of this society will never be fully attained until its influence shall have been so exerted, as to bring dental practice under the protection of law and order, as has been done in the state of Alabama, by the wisdom of its legislature. But this auspicious event in that favored state, was brought about, mainly, I believe, by members of this national society, acting under the influence which this body has been able to exert, through its annual meetings, and its periodical journal. Every additional state which can be induced, by the members of this society, within its borders, to adopt the same enlightened policy, which has given the death blow to dental empiricism in the above named state, will add strength to the movement; and it is not too much to hope, from the advancement of the past thirty years, that the next thirty will bring all, or nearly all the states of this republic, into the same fortunate and much to be desired condition. And if this shall be the desirable result, it must be mainly owing to the vigorous exertions of this association, and of state societies organized for local purposes.

To effect this object, I can imagine no more successful measure, than to secure the interests of some of the influential members of our state legislatures; or if some members from our own profession could be elected, such practitioners as would be able to subserve the general objects of legislation, as well as the object we have in view, our wishes might be accomplished.

Physicians are frequently sent to our legislative bodies, with very great propriety; and why not dental surgeons, in order that all the great interests of society may be properly represented? for, in this case, it could be easily shown, that the *public* and not *individual* good was our great object.

The Journal is our most effective, direct and universal medium of professional information; all the learned, reputable and useful professions or callings have their *periodicals;* this is, indeed, an *age* of periodical literature, and our Journal has been acknowledged to keep pace with the *spirit of the age;* and I think it should be one of the first objects of our present session to endeavor to adopt the measures necessary to impart new vigor, and give increased circulation to this valuable work. We are certainly able, among the members of this association, to constitute an editorial corps, which shall do honor to the association, to the profession, and to the science of dental surgery. But I would here remark, that there are but very few of those who feel pleasure in reading the Journal, that are aware of the time and labor that is bestowed upon it by its editors. So far, their labors have been gratuitously given, for the general good of the profession, prompted by the noble desire to elevate the standard of professional excellence, particularly in this country. But we have no right to expect this always to be the case; therefore some means ought to be devised, by which the Journal could be made to yield to the editors some compensation for their labors; and, besides the pleasure they feel in disseminating useful information, and the interest they have in the general welfare of the profession, that they might also otherwise feel encouraged to devote their time and talents to the work. All that are engaged in the profession know that "time is money;" and it may so happen, that that very time is all the means at their command to support their families, and ought, therefore, to be compensated; and, as an act of justice, we ought, if possible, to adopt measures by which the Journal can be made to yield a handsome profit over and above the expenses, and that profit should go to reward editorial drudgery.

It ought to be remembered, that the more we can extend the circle of correct professional information, the more good and efficient dentists we shall have among us, and although all may not become members with us, the community at large will receive the benefit resulting from the experience of the best and ablest practitioners in the art, which will be diffused through the pages of the Journal, and which has already been felt by hundreds of our professional brethren, in all parts of the country, many of whom have become fellows and associates of the American Society of Dental Surgeons.

I have been informed, that the present price of the Journal prevents some from taking it; and that there are others scattered through the country, who are unacquainted with the terms and requisitions of the society in order to become members, who are out of the way of the circulation of the Journal, and who need correct information, both in relation to that, and to the condition and state of the society. Those who have always resided in the cities know but little of the great want of general information on subjects connected with our professional practice; nor are they aware of the pecuniary embarrassment and difficulties with which dental operators have to contend in the interior towns and villages. Therefore, the wants of the people, as well as the profession abroad, require a helping hand from this society.

These considerations, with others, which time will not allow me to enumerate or enforce, will, I trust, incline our hearts to regard all those whose welfare has been entrusted by its founders to the fostering auspices of this society.

Before I dismiss the subject, on which I should be glad to dwell at much greater length, I must ask your indulgence while I say that, among the many reasons for mutual congratulation, we have at this time connected with the operations of this society, the elevation of the principles of our art to the dignity of a science, by embodying them permanently in our Journal, deserves particular notice in this place. It has now reached the completion of its *fifth volume.* Thirty years ago the man would have been thought insane, who should have predicted that the dental profession in the United States would become so respectable, and so extensive in 1845, as to have sustained a periodical which should have issued five large and beautiful octavo volumes, with a fair prospect of perpetuating the work for the benefit of posterity.

One thing is evident, that our successors in dental practice, will either continue this or some kindred work, or acknowledge, by their neglect of such a publication, that the present period has been really the *Augustan age of Dental History.*

Either alternative will confer sufficient honor on those talented and spirited individuals who have so benevolently embellished the pages of our Journal with the vigorous efforts of their minds, as well as on those who have sustained the publication from year to year by their liberal contributions.

For my part, I regard it as a high honor to have been connected with the profession of dental surgery, at a period, the achievements of which are thus *distinguished* and *enviable*—especially when I reflect that the actors in these achievements have been my *friends* and *companions.*

As one of our patriots said of the constitution of the United States—"*It must be preserved,*"—so will every true lover of our art, and of its usefulness in society, say—*The American Journal and Library of Dental Science* MUST BE PERPETUATED.

But whether it shall be continued in its present form, and at its present price—or whether it shall be reduced in the quantity of reading matter, and issued more frequently at a lower rate, are matters to be submitted to the wisdom of the society. There can be no doubt but that in its present form much valuable matter remains with many *unread*, which would not be the case, to the same extent, if smaller numbers were issued more frequently.

There is one other matter which I think might be acted upon with very great advantage, and which would be likely to secure to us legislative aid; and that is, if a committee could be appointed to draft and offer good and wholesome rules which the whole society could approve, by which dentists should be governed in their practice—setting forth the requisite qualifications, to entitle them to public confidence—so as to place empiricism on the same footing, in relation to dental practice, that it now has in general surgery and medicine. Our legislative bodies would at once see the incalculable benefit of it, and would, I believe, enact such laws as would protect the public from the most flagrant abuses that ever disgraced individuals in any calling—for all who are in general practice can testify, that our state prisons have many inmates in them, for incomparably less crimes than are daily perpetrated by the Crawcourean host of wholesale swindlers on the honest industry, and unsuspecting simplicity of the dupes to their artifices. And the extent of these abuses are only known to the regular practitioners, who are afterwards called upon to remedy, or correct, so far as they can, the evils thus committed, which unfortunately oftentimes are beyond their power.

A well drafted and well digested set of rules, regulating the admission of men to dental practice, and governing them in their practice after they were so admitted, would do more good to the profession, and to the community at large, than any other step that could be possibly taken. I would, therefore, if it should meet the views of the society, cordially recommend it to be acted upon at the present session. And if the legislature will do nothing more than merely to regulate the conditions by which members shall be admitted to practice—although trifling as the regulation might seem—it would serve, at least, to draw a line of distinction, which the public would understand, between the regular members of the profession, and the quacks who disgrace it. This would be an important point gained, because it would enable the public to form some conjecture as to the qualifications of those they employ, and to whom they entrust organs so important to their health, peace, and comfort—to say nothing of their inestimable value to beauty and to personal attraction.

The position which this society has assumed in the public eye, requires of it that something shall be done for the general welfare; and I am convinced that the greatest obstacle in the way of the general usefulness of the profession is found in the degradation both of professional charges and dental practice, by men who work for *nothing*, because their work is worth *less* than nothing. While such men are abroad, more numerous and pestilential than the frogs of Egypt, it is vain to expect that respectable and well-qualified dentists can obtain respectable support in the towns and villages of our country. It will be only in a few large cities that good practitioners can sustain themselves. Unless some measures are adopted, the character of the profession will be more and more degraded in the eyes of the community—whilst ignorant mountebanks and swindlers will perpetrate, unchecked, their miserable and unprincipled malpractices on the easy credulity of man, but more especially and more successfully on womankind.

There is a means by which much good might be done by the members of this association, in enlightening the public mind as to their real and true interests; and that is, by having embodied, in a few plain precepts, the general principles of dental practice—with short but comprehensive rules for the government of individuals, of all ages, in the management of their teeth. A small pamphlet containing precepts of example and instruction, issued from the united wisdom and experience, and by the authority of this society, would do much in governing people as to what they should have done, and at the same time cautioning them as to whom they should employ to do it.

If the principles and practice of dental surgery could be thus brought out, in a number of precepts, so condensed, as to occupy but little space, as it would at once be seen that the object was public good, benevolent and philanthropic editors would no doubt republish them for the good of their patrons, and thus thousands of copies would obtain immediate circulation, to the great advantage of suffering humanity. And as the expense would be but small, the members might circulate them freely among all classes of people, and thereby set forth the *uses* and *abuses* of dental practice, of which the public at large, for want of correct information, are almost wholly ignorant.

It appears to me that such a thing, under the sanction and approval of the society, would do more to suppress the quackery and imposture that now prevail, to a most alarming degree, than any other practicable plan of equally easy execution.

These precepts might, with propriety, embrace—what should be the character and qualifications of the dentist to be employed in the care and management of the teeth—the care that should be bestowed on the first as well as on the second set of teeth—the anatomical structure and chemical analysis of the teeth, in order to show what would prove hurtful to them—the operations which they require, and the time for these operations, and the means to be employed in performing them, and the materials to be used—the bad effects of having, and the total want of honesty in using improper materials for stopping teeth—and the ruinous effects of using improper substances for cleaning the teeth—and proper directions for their care and preservation. These precepts or rules might be extended to as many as would express fully our united views in relation to the subjects embraced.

Having, in Europe and in this country, passed through thirty years of unparalleled success, I will, more especially for the benefit of the younger members, in a few words, state what has been my experience as to the causes of failure and success in dental practice.

During that period I have known some that have commenced with all the advantages that the most favorable circumstances could throw around them; and have seen them for a time followed with the most flattering success—but being too much flattered by success, they were, in their vain imaginations, soon elevated above their true positions; and then, rather than stoop to the calls of professional duty, in their lofty elevation, they struck against the unyielding bars of pride and arrogance; beneath which they fell, and from the fall never rose again.

Others, beginning in the same way, after a few years of success, have considered that dentists were rather lightly esteemed by the aristocracy of our land of equality, have been dissatisfied and changed their occupations; and although some of them have for a time been successful in their new pursuits, (and in one instance, in particular, one of my early friends, an exceedingly clever man, William S. Parrott, was at one time possessed of immense wealth, and rose to high official distinction under our government, and was also an eminent merchant and banker in the city of Mexico,) yet, as far as I am acquainted, they have all failed of the accomplishment of the objects of their pursuit.

Others, again, have taken up the profession, and have attended to the practice as a kind of menial occupation, which they only performed in order to live; but from their family, education, and consequence, always considered themselves far above it, and felt their dignity touchingly invaded by the drudgery of professional labor; thereby making their professional character altogether inferior and subservient to that by which they wished to be known and regarded among men.

Others, again, without knowledge, honesty, or truth, have, by imposture, fraud, and intrigue, imposed upon the world, and have obtained the means of living for a time in the most corrupt and licentious practices, until their excesses put a period to their earthly, as well as to their business career.

There are others who have been well instructed, and have possessed a high degree of professional skill and dexterity in their operations, but have been so totally void of truth and integrity—betraying the confidence of their patients, and disappointing them in their expectations—that they have wholly failed of success; bitterly complaining at the same time that their merits were not discovered, and their learning, talents, and skill were not known and fully appreciated.

Those that have succeeded, and the proportion is small of the whole number, are they that, with steadiness of purpose at the outset, made themselves acquainted with the theory and practice of their art—with its requirements and duties—with its obligations and responsibilities—and never swerved from them; who, by an undeviating course of integrity and truth, secured the confidence of their friends and patrons, and never betrayed or lost it—who never considered any professional claim, whether made by the high or the low, the rich or the poor, as beneath their notice—who felt proud of the profession that was giving them distinction, instead of believing that they were honoring the profession by their transcendant talents and professional grandeur—who, faithfully and honestly, performed every duty; feeling that the services rendered by them were more than equivalent to the money received—who have pride of character and moral worth enough, not to descend to the vile and dishonest practices of charlatans and robbers, because they might and because the Crawcours did make money by them.

I therefore would earnestly recommend that you, my respected young friends, adopt in your practice, and carry out in your lives those principles which have been found so eminently successful with those that have observed them; and, although you may meet with many obstacles at the beginning, by perseverance you will overcome them, and ultimately attain to the first walk in the profession in which you are engaged—securing, day by day, and year by year, the confidence, respect, and friendship of the community in which you live, as well as, more especially, that of your professional acquaintance and patrons.

I wish, farther, to be permitted to make a remark or two on a matter of business that necessarily comes before us at our annual meetings—that is, in relation to the election of members. I do not approve of the practice of any who use mercury or quicksilver as an ingredient for stopping teeth, and would not privately recommend any one to go to such. Why, therefore, should they have the sanction and influence of the society to assist them in carrying out what we know, and what they admit, is not the best practice in dental surgery?*

What should we think of a surgeon, who, on being applied to treat a wounded hand, would say—"I have a remedy that will cure it, but it is expensive. I will, therefore, dress it to-day, *without the least pain*, with a little cooling adamantine plaster, which (although it will poison a little) will not cost much." And, in consequence of which, the man, in a short time, loses his hand; and the surgeon knew it most likely would be so, when he applied the plaster. Could we suppose that any medical society would retain such a man as a member. And with such a man, I rank all who say that "mercury and silver, or zinc and mer-

* The admission of Doctor Baker yesterday, who is not only one of the oldest, but one of the most highly respectable dentists in the country, who has not only used it, but has thought so well of it as to recommend it to others, is beyond price to us in the effort we are now making to suppress its use. He admitted before you all that it is *"a bad filling"*—that it is *"the worst kind of filling"*—that it is a *"nasty filling"*—*"and that it is the worst thing in the world to fill teeth with, except as a filling for the mere shell of a tooth that will bear nothing else."* Such an admission from such a man as Dr. Baker should make the face of every honorable man blush to own that he uses it, or to give the slightest encouragement, or in any way sanction its use.

cury, is as good as gold, and half the price," and who have been known to take out gold stoppings, and insert their amalgam instead. Such men, instead of dentists or dental surgeons, should be called succedaneum or adamantine cement tooth plasterers—for they deserve no better name.

If it is an advantage to the honest and conscientious practitioner to belong to, and to obtain the certificate of the society, in order to enable him to secure the confidence of the public, it is also an advantage to the dishonest and unprincipled practitioner, in order to secure for him the same confidence, to enable him to carry out his impostures. The public, in both cases, relying upon the integrity of the society for the character of the individual who bears its name. And thus, instead of correcting the existing evils, we should be adding to them, by having members who would not practice in accordance with the most highly approved principles of dental practice, as known, understood, and approved by this society. And if it permits the introduction of such men among its members, I must beg leave most respectfully to withdraw from the body, for I could not conscientiously sign a certificate of the society to be used by one to whom I would not give a private letter, recommending his practice. And I do not know by what rule of justice, we could give a man a passport to the confidence of strangers, that we would not introduce and recommend to our friends.

I would not associate with me in private practice any man who would use, or recommend to be used, an amalgam of mercury and silver, for stopping teeth, under the imposing names of succedaneum, mineral paste, diamond cement, adamantine cement, &c., &c. And the men who use this under these names, and at the same time say that crude mercury does not enter into its composition, I unhesitatingly say do not speak truth; and I challenge the whole catalogue of them to come out, and defy them to prove what they assert in private, and also in their public advertisements, wherein they shamelessly and unblushingly say, that it is not what I here most positively declare it to be—a base compound, chiefly composed of mercury and silver, or zinc, wholly unfit for the exceedingly nice and important operation of stopping teeth. It will, therefore, be very necessary that we examine carefully into the character and intentions of those whom we may admit as our fellows and associates. And I cannot better conclude these remarks, which have already gained greater length than may be deemed profitable, than by using the words of our late venerable, respected, and revered President—the wisdom and truth of whose sayings, we are, in our experience, daily proving; and in relation to this particular point, said at our first meeting—"Gentlemen, if you would make your society respectable, and would have it esteemed so by the world, do not be hasty in electing your members, but be careful and judicious in selecting those whom you would admit into your ranks."

